# Complete plastid genome of *Gracilaria bailiniae* (Rhodophyta) and phylogenetic analysis

**DOI:** 10.1080/23802359.2018.1535844

**Published:** 2018-11-21

**Authors:** Tao Liu, Shouguo Yang, Xiangyu Wu, Wei Zhou Chen, Jing Zhang, Xianming Tang

**Affiliations:** a College of Marine Life Sciences, Ocean University of China, Qingdao, Shandong Province, People’s Republic of China;; b Hainan Academy of Ocean and Fisheries Sciences, Haikou, Hainan Province, People’s Republic of China;; c Marine Biology Institute, Shantou University, Shantou, Guangdong Province, People’s Republic of China;; d Qilu University of Technology (Shandong Academy of Sciences), Jinan, Shandong Province, People’s Republic of China

**Keywords:** *Gracilaria bailiniae*, phylogenetic analysis, plastid genome

## Abstract

The complete *Gracilaria bailiniae* plastid genome was determined and analyzed in this work. It had a circular mapping molecular with the length of 185,129 bp and contained 231 genes including 193 protein-coding, 3 rRNA, 1 tmRNA, 29 tRNA genes, and 5 unidentified open reading frames. Phylogenetic analysis showed that *G. bailiniae* clustered together with *Gracilaria chorda* and *Gracilariopsis lemaneiformis*. The complete plastid genome provided in this work would be useful for elucidation of *Gracilaria* evolution.

*Gracilaria bailiniae* (J.F. Zhang & B.M. Xia) J.F. Zhang & B.M. Xia (Gracilariaceae, Rhodophyta) is a marine red alga. *Gracilariopsis bailiniae* J. Zhang & B.M. Xia is the basionym of *Gracilaria bailiniae*. As an agar-producing seaweed (Rabanal et al. 1997), efforts have been directed towards the development of culture techniques and the improvement of agar quality (Castaños and Buendia 1998). However, there has been no genomic studies on *G. bailiniae*.

Herein, we reported and characterized the complete *G. bailiniae* plastid genome (MF372957). One *G. bailiniae* individual (specimen number: 2016050140) was collected from Shantou, Guangdong Province of China (21°24′26′′ N, 111°13′7′′ E) and stored at −80 °C in the Culture Collection of Seaweed at the Ocean University of China for DNAs isolation. Paired-end reads were sequenced by using Illumina HiSeq × Ten system (Illumina, San Diego, CA). Approximately 9 Gb of paired-end (150 bp) sequence data were randomly extracted from the total sequencing output and used as input for NOVOPlasty (Dierckxsens et al. 2017) to assemble the plastid genome. The plastid genome of *Gracilaria chorda* (GenBank accession number: NC_031149) was used as the seed sequence. The tRNA genes were identified by using tRNAscan-SE Search Server (Schattner et al. 2005). Other regions were annotated from the *G. chorda* plastid genome by using Geneious R10 (Biomatters Ltd, Auckland, New Zealand). The nucleotide sequences were aligned by using MAFFT (Katoh et al. 2002). Concatenated alignments were generated and poorly aligned regions were removed by using the Gblocks server (http://phylogeny.lirmm.fr/phylo_cgi/one_task.cgi?task_type=gblocks) (Castresana 2000). The phylogeny based on the complete plastid genome shared by Gracilariaceae species was inferred from the ML search and ML bootstrap analysis using RAxML (Stamatakis 2006); bootstrap probability values were calculated from 1000 replicates and *Rhodymenia pseudopalmata* (KC875852) served as the out-group.

The complete *G. bailiniae* plastid genome is a circular DNA molecule with the length of 185,129 bp. The overall AT content of the complete plastid genome was 62.1%. The plastid genome contained 231 genes, including 193 protein-coding, 3 rRNA, 1 tmRNA, 29 tRNA genes, and 5 unidentified open reading frames. Phylogenetic analysis showed that *G. bailiniae* clustered together with *Gracilaria chorda* and *Gracilariopsis lemaneiformis* (Figure 1) which indicated the phylogenesis classification of *G. bailiniae*. The determination of the complete plastid genome sequences provided new molecular data to illuminate the *Gracilaria* evolution.

**Figure 1.**
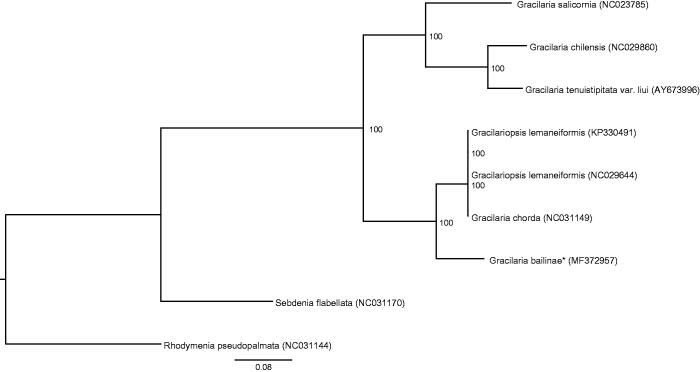
Phylogenetic tree (maximum likelihood) based on the complete plastid genome sequence of Gracilariaceae. The numbers along the branches are RAxML bootstrap supports based on 1000 nreps (<70% support not shown). The asterisks after species names indicate newly determined mitochondrial genomes.

## References

[CIT0001] Castaños M, Buendia R 1998 Farming techniques for seaweeds. SEAFDEC Asian Aquaculture. 20:14–19.

[CIT0002] Castresana J 2000 Selection of conserved blocks from multiple alignments for their use in phylogenetic analysis. Mol Biol Evol. 17:540–552. 10742046 10.1093/oxfordjournals.molbev.a026334

[CIT0003] Dierckxsens N, Mardulyn P, Smits G 2017 NOVOPlasty: de novo assembly of organelle genomes from whole genome data. Nucleic Acids Res. 45:e18. 28204566 10.1093/nar/gkw955 PMC5389512

[CIT0004] Katoh K, Misawa K, Kuma K, Miyata T 2002 MAFFT: a novel method for rapid multiple sequence alignment based on fast Fourier transform. Nucleic Acids Res. 30:3059–3066. 12136088 10.1093/nar/gkf436 PMC135756

[CIT0005] Rabanal SF, Azanza R, Hurtado-Ponce A 1997 Laboratory manipulation of *Gracilariopsis bailinae* Zhang et Xia (Gracilariales, Rhodophyta.). Bot Mar. 40(1-6):547–556.

[CIT0006] Schattner P, Brooks AN, Lowe TM 2005 The tRNAscan-SE, snoscan and snoGPS web servers for the detection of tRNAs and snoRNAs. Nucleic Acids Res. 33:W686–W689. 15980563 10.1093/nar/gki366 PMC1160127

[CIT0007] Stamatakis A 2006 RAxML-VI-HPC: maximum likelihood-based phylogenetic analyses with thousands of taxa and mixed models. Bioinformatics. 22:2688–2690. 16928733 10.1093/bioinformatics/btl446

